# An analysis of facial blood perfusion in patients with peripheral facial palsy

**DOI:** 10.1097/MD.0000000000049334

**Published:** 2026-07-03

**Authors:** Jie Wang, Yang Li, Jiandong Li

**Affiliations:** a Department of Otolaryngology, Electric Power Teaching Hospital of Capital Medical University, Beijing, China; b Department of Otolaryngology, Beijing Chaoyang Hospital, Capital Medical University, Beijing, China; c Department of Otolaryngology, The First Largest Otolaryngology Hospital, Beijing, China.

**Keywords:** blood perfusion, laser speckle contrast imaging, peripheral facial palsy

## Abstract

This study aimed to investigate the value of laser speckle contrast imaging in detecting facial blood perfusion in patients with peripheral facial palsy (PFP); to establish the evaluation criteria for facial blood perfusion; and to clarify the relationship between facial microcirculatory status, facial nerve function, and facial blood flow changes in PFP patients. Thirty healthy controls and 132 PFP patients receiving treatment at our hospital were chosen for laser speckle contrast imaging, in order to quantify their facial blood perfusion at the following 4 sites: the periocular region, the middle cheeks, the corners of the mouth, and the nasolabial fold. Blood perfusion values (BP) were measured for these sites, and the rate of blood flow changes was calculated as follows: R = (BP_affected side_-BP _healthy side_)/BP_healthy side_. The changes in blood flow rates in the periocular region and cheeks were significantly different between patients with severe peripheral facial paralysis (PFP) and healthy controls. However, no significant differences were observed at these sites between patients with Ramsay Hunt syndrome and those with Bell’s palsy. In patients with moderate and severe PFP, the rate of blood flow changes in the periocular region was lower compared to healthy controls. Additionally, patients with severe PFP showed lower rates than those with moderate PFP. A lower Sunnybrook score in PFP patients correlated with a smaller rate of blood flow changes in the periocular region. For patients with Bell’s palsy, blood flow rate changes in the periocular region at two- and four-months post-surgery were significantly different from pre-surgery levels. There was also a significant difference between the rates at two and four months. In patients with Ramsay Hunt syndrome, the rate of blood flow changes at two months post-surgery did not significantly differ from pre-surgery. No significant difference in blood flow rate changes at two months post-surgery was observed between patients with Bell’s palsy and Ramsay Hunt syndrome. Facial blood perfusion decreased with the severity of facial nerve injury in PFP patients, regardless of the etiology. Postoperative improvements were significant in Bell’s palsy, but slower in Ramsay Hunt syndrome.

## 1. Introduction

Peripheral facial paralysis is a common clinical disease, which is caused by damage to the facial nerve nucleus and nerves below it, resulting in facial muscle paralysis on the affected side. It is known that microcirculation disorders of the temporal bone of the facial nerve are present in patients with facial paralysis. Will this microcirculation disorder affect various branches of the facial nerve? And how to detect the microcirculation of various branches of the facial nerve?

In clinical practice, tissue microcirculation function is often reflected by measuring tissue blood perfusion flow. Laser speckle contrast imaging (LSCI), as a non-scanning full field imaging technology, has advantages such as simple equipment, noninvasiveness, no need for injection of contrast agents, fast imaging speed, high resolution, and long-term continuous measurement.^[1–3]^ It has been widely used to measure microcirculation blood flow parameters such as blood vessel diameter, blood flow velocity, blood perfusion, and blood flow density in tissues and organs such as the retina, skin, and brain.^[4–7]^ It provides an effective technical means for analyzing the structure, function, and metabolism of tissues and organs, thereby facilitating disease diagnosis, intraoperative monitoring, and pathogenic mechanism research.^[8–11]^

We compared the differences in blood flow perfusion in 4 facial regions among patients with Bell palsy, those with Hunt’s syndrome facial paralysis, and normal individuals, as well as in the periocular region before and after surgery. This was done to analyze the selection of monitoring sites for facial blood flow perfusion and to determine whether there are differences in blood flow perfusion between different causes, degrees of lesions, and preoperative and postoperative peripheral facial paralysis.

## 1. Materials and methods

### 1.1. Laser speckle contrast analysis

Laser speckle contrast analysis was performed using the PeriCam PSI System (Perimed, Sweden). The raw data were processed with the help of a computer to generate blood perfusion images. Blood perfusion was measured using the 70-mW laser diode in the PeriCam PSI System, at a laser wavelength of 785 nm and a camera resolution of 1388*1038 pixels. The raw tissue blood perfusion data were then subjected to accelerated processing using a computer.

### 1.2. Subjects

Thirty healthy controls and 132 peripheral facial palsy (PFP) patients treated at our hospital were selected. Based on the Sunnybrook score, they were divided into the severe PFP group (0–33 points) and the moderate PFP group (33–70 points) and received LASCA. Four sites were chosen: the periocular region, the middle cheeks, the corners of the mouth, and the nasolabial fold. Blood perfusion values (BP) were measured at these sites, and the rate of blood flow changes was calculated using the formula [R = (BP_affected side_-BP _healthy side_)/BP_healthy side_].

Exclusion criteria: 1. recurrent facial palsy, bilateral facial palsy, facial neuroma, and PFP caused by facial nerve injury; 2. Sunnybrook score > 70^[12]^; 3. history of skin diseases, such as facial acne and dermatitis.

### 1.3. Research method

The system was calibrated before the formal measurement. The monitoring result of the standard calibration solution at 23°C was 250 ± 5 PU. The measurement in PFP patients was conducted at a room temperature of 23°C, under natural lighting conditions. The patients were first given 5 minutes to get acclimatized to the environment and were asked to maintain a supine position and to remain motionless during the measurement. The measurement continued until about 30 seconds after the test curve stabilized.

Statistical analyses were conducted using SPSS 22. The basic clinical features of the patients were analyzed by descriptive statistics. Measurements obeying a normal distribution with homogeneity of variance were analyzed by 1-way analysis of variance (ANOVA). Skewed data or data with heterogeneity of variance were analyzed by nonparametric test. Data with a bivariate normal distribution were analyzed using Pearson product-moment correlation coefficient. *P* < .05 was considered to be statistically significant.

## 2. Results

### 2.1. Blood flow differences in severe PFP patients

**Figure 1.**
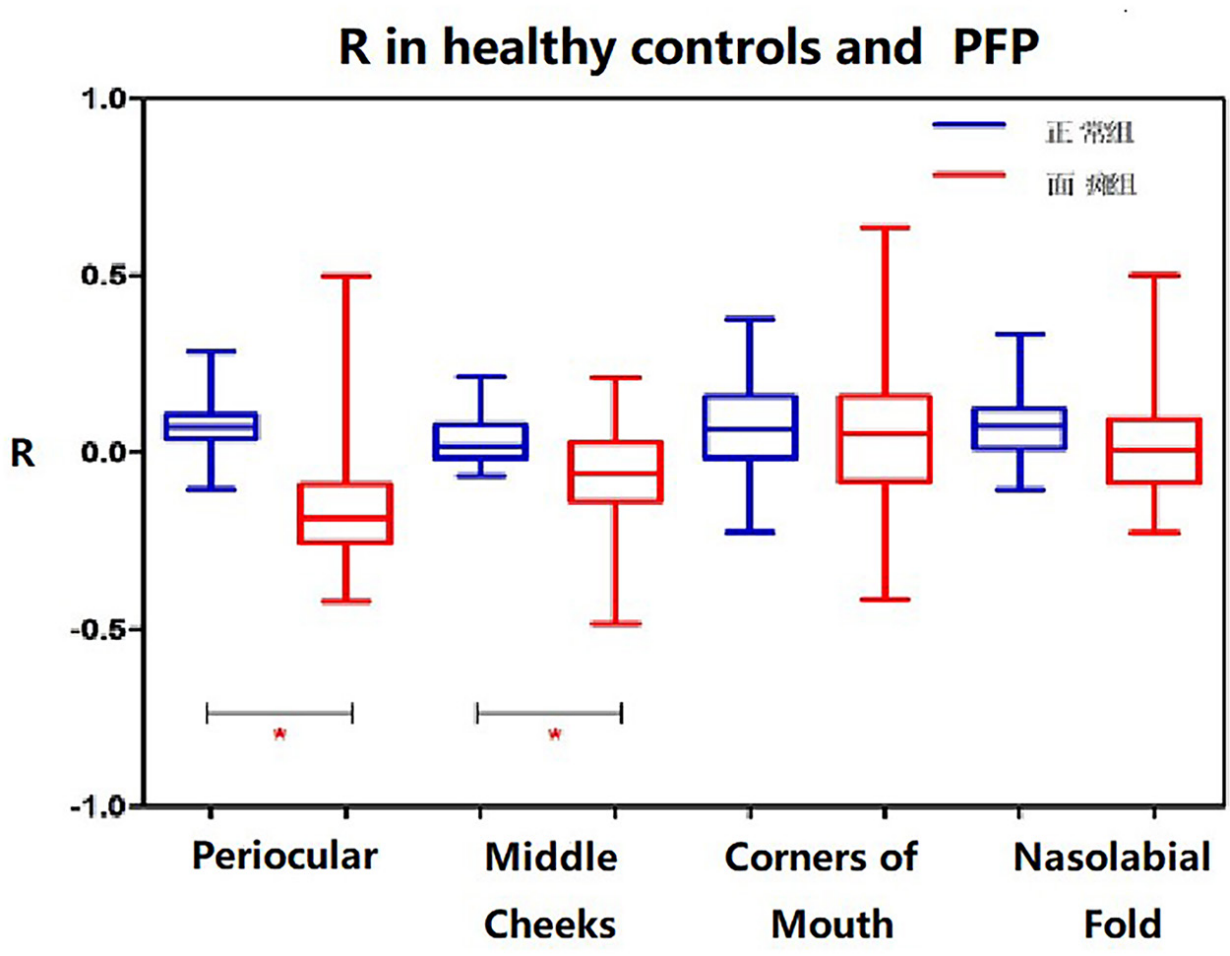
R in healthy controls and patients with severe PFP. The blue line represents the normal group, while the red line represents the facial paralysis group.*represents *P* < .05.

LSCI was employed to measure BP values in healthy controls and 67 patients with severe PFP (including 29 patients with Ramsay Hunt syndrome and 38 patients with Bell palsy). The rate of blood flow changes at each site of measurement was estimated using a formula and was compared at each site of measurement between the healthy controls and patients with severe PFP. Figure 1 shows the rates of blood flow changes in the periocular region and cheeks were significantly different between the patients with severe PFP and the healthy controls.

### 2.2. Blood flow variation by etiology in severe PFP patients

**Figure 2.**
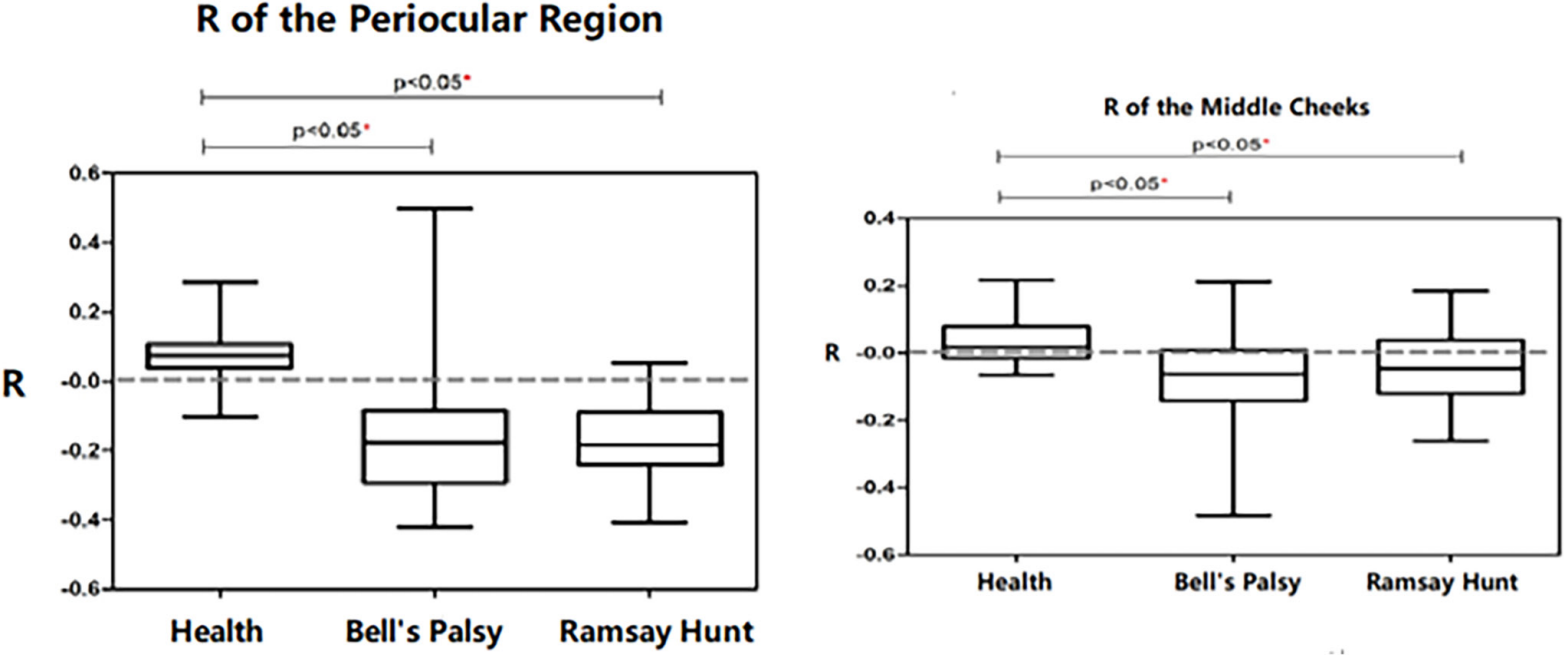
R of the periocular region and R of the middle cheeks.

Patients with severe PFP were divided into the Bell palsy group and the Ramsay Hunt syndrome group, based on etiology. They were subdivided into 4 groups based on the site of measurement. The rates of blood flow changes at each site were compared between the healthy controls and the patients with Bell palsy and Ramsay Hunt syndrome. In the periocular region and the cheeks, the rates of blood flow changes in patients with Bell palsy and Ramsay Hunt syndrome were significantly different from those of the healthy controls. However, at these sites, no significant difference was observed between the patients with Ramsay Hunt syndrome and those with Bell palsy (Fig. 2). In the corners of the mouth and the nasolabial fold, there was no significant difference in the rate of blood flow changes between the 3 groups.

### 2.3. Blood flow changes in periocular region based on sunnybrook score

**Figure 3.**
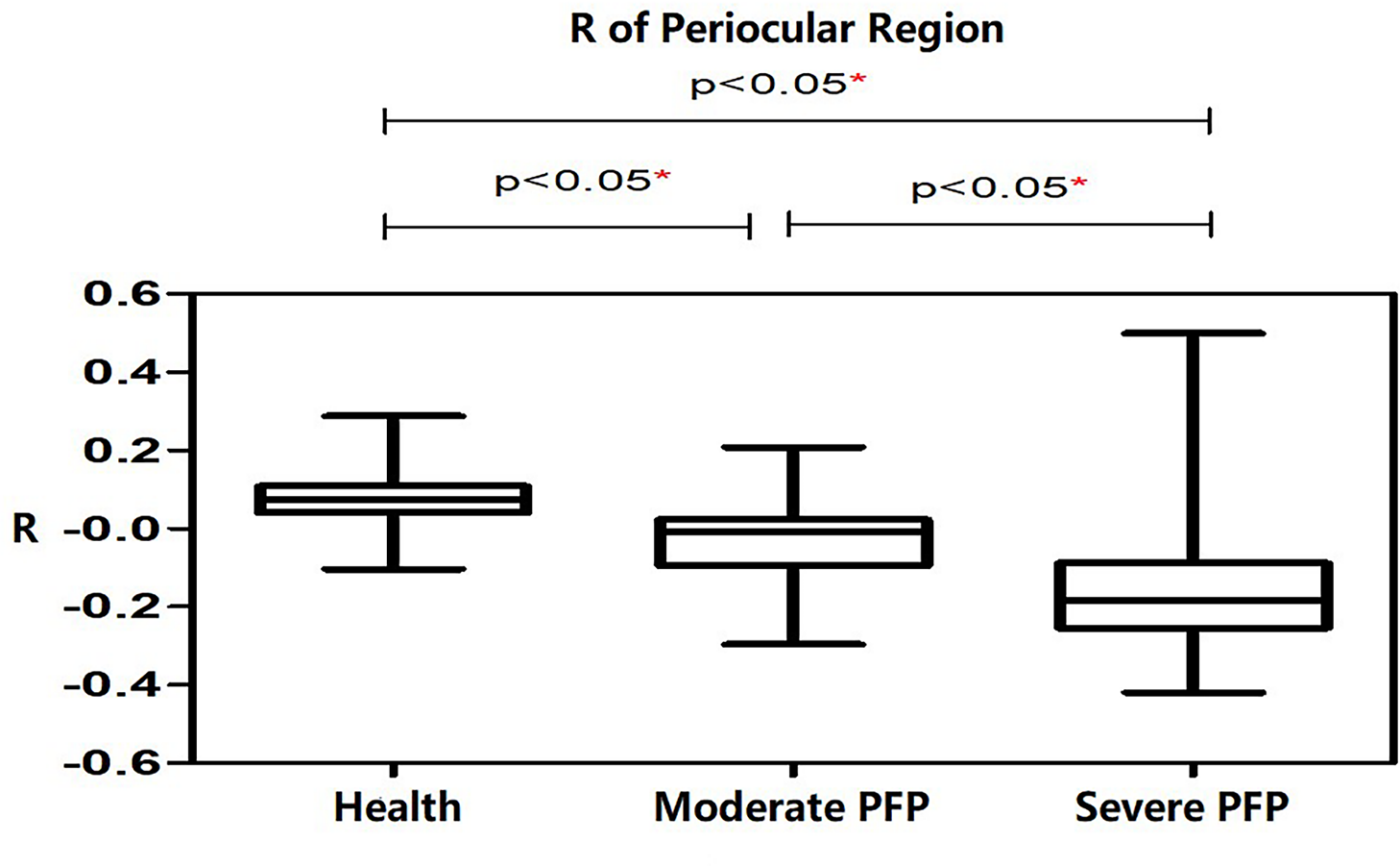
R of the periocular region with degrees of lesions.

Based on the Sunnybrook score, the PFP patients were divided into the moderate PFP group (n = 20, with an average Sunnybrook score of 48.76) and the severe PFP group (n = 67, with an average Sunnybrook score of 13.46). A comparison was made between these 2 groups versus the healthy controls, regarding the rate of blood flow changes in the periocular region. Figure 3 shows a significant difference was observed between the moderate PFP group and the healthy controls in the periocular region. Such a difference was also observed between the severe PFP group and the healthy controls. The difference between the moderate and the severe PFP groups was also of statistical significance. The correlation between the Sunnybrook score and the rate of blood flow changes in the periocular region was analyzed. The Pearson correlation coefficient *R* = 0.389, *P* < .05 (two-sided), indicating that the Sunnybrook score was positively correlated with the rate of blood flow changes in the periocular region.

### 2.4. Periocular blood perfusion changes post facial nerve decompression

**Figure 4.**
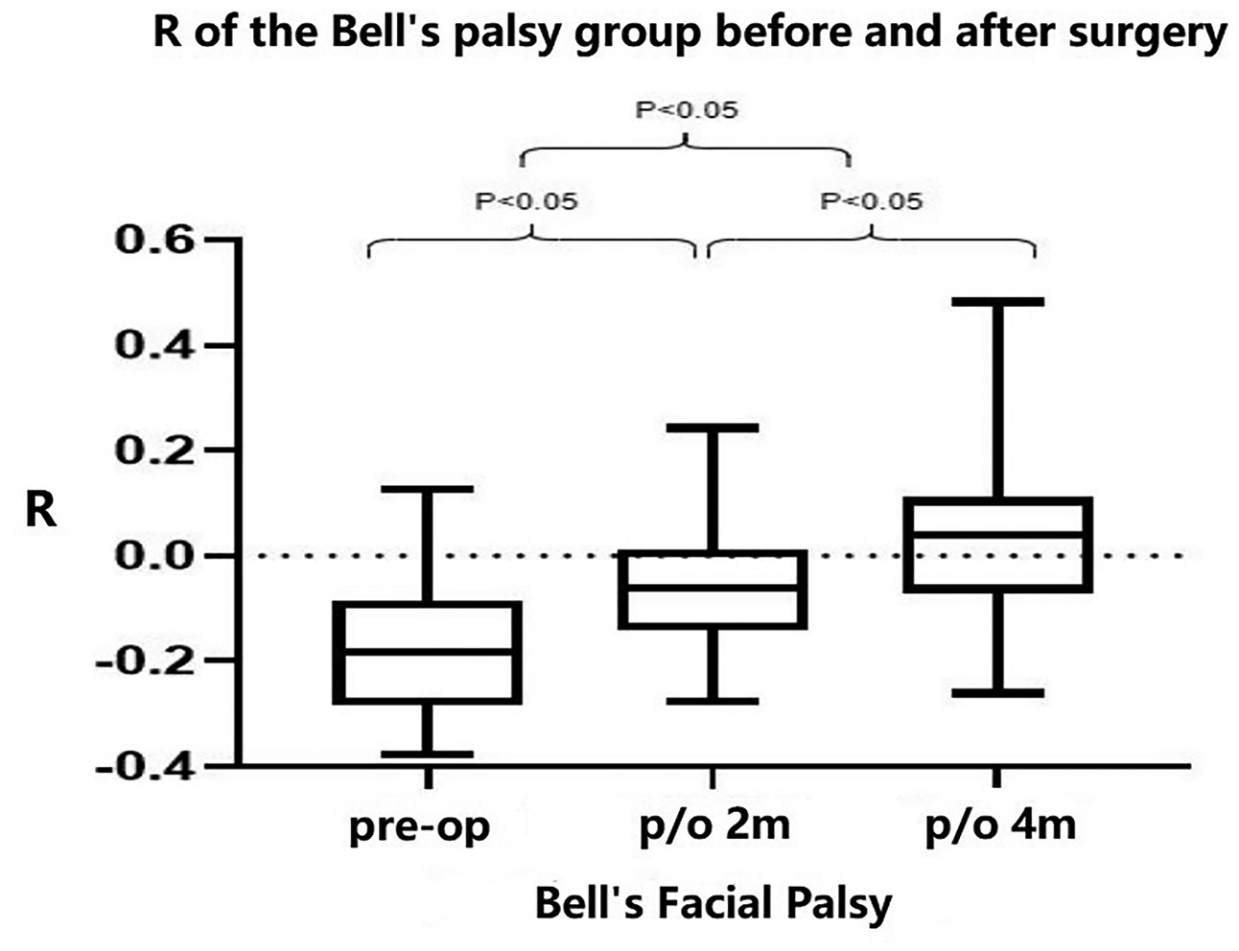
R of the Bell palsy group before and after surgery in periocular region.

Forty-five severe PFP patients who had received subtotal facial nerve decompression were divided into the Bell palsy group and the Ramsay Hunt syndrome group, based on etiology. Blood perfusion changes in the periocular region were analyzed before and after surgery. Among patients with Bell palsy, the rates of blood flow changes in the periocular region at 2 and 4 months after surgery were significantly different from those before surgery. Moreover, a significant difference in the rate of blood flow changes was also observed in the periocular region between 2 and 4 months after surgery (Fig. 4).

### 2.5. Blood flow changes in periocular region post-surgery: ramsay hunt syndrome versus Bell Palsy

**Figure 5.**
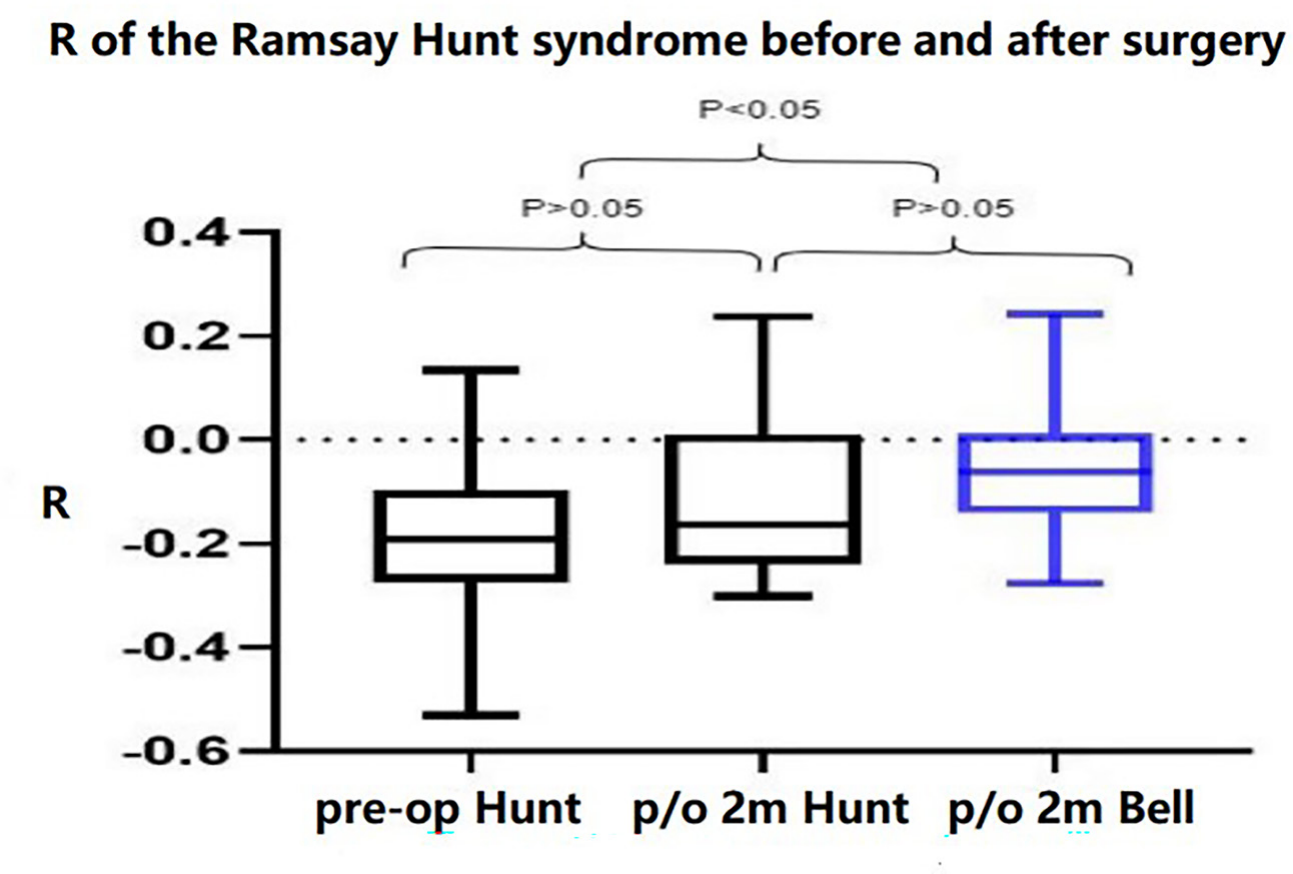
R of the Ramsay Hunt syndrome before and after surgery in the periocular region.

In those with Ramsay Hunt syndrome, the rate of blood flow changes in the periocular region at 2 months after surgery was not significantly different from that before surgery. We further compared the rate of blood flow changes in the periocular region at 2 months after surgery between patients with Bell palsy and Ramsay Hunt syndrome and found no difference of statistical significance. However, the value was significantly different between the patients with Bell palsy at 2 months after surgery and those with Ramsay Hunt syndrome before surgery (Fig. 5).

## 3. Discussion

Both Bell palsy and Ramsay Hunt syndrome are related to swelling of the facial nerve,^[13]^ as has been confirmed by either facial nerve decompression or MRI.^[14]^ Lesions involving the facial nerve, especially the labyrinthine segment, can cause a loss of the innervation and nourishing effects of the distal facial nerve regarding the muscles involved in facial expressions. This further results in a reduction in microcirculatory perfusion of the facial skin. Few anatomical and histological analyses about the muscles of facial expressions affected by PFP have been conducted so far. In the case of denervated skeletal muscles of the extremities, a dramatic reduction in microvessels is a common occurrence, apart from the long-term involvement of the muscle fibers. Borisov found that the number of capillaries related to the number of muscle fibers, i.e., the capillary-to-fiber ratio, decreased by 88% during the first 7 months after denervation and then slightly declined at a much lower rate during the next 11 months of observation to 10% of the capillary-to-fiber ratio in normal muscle.^[15]^

LSCI has rarely been applied to measure facial blood flow, especially in PFP. Cui^[16]^ showed that, compared with the normal group, the periocular blood perfusion on the affected side decreased significantly in patients with Bell palsy, which is different from the situation on the healthy side. Besides, the more severe the injury, the greater the difference between the 2 sides. The facial microcirculatory status could reflect the stage of Bell palsy. The above study confirmed the changes in facial skin blood flow in the periocular region of patients with Bell palsy. Weizheng Zhong^[17]^ investigated the effect of manipulative acupuncture monitored by LSCI on improving facial blood perfusion in patients with severe Bell palsy. Laser speckle technology can capture facial blood perfusion and monitor the changes during acupuncture. LSCI is significant for acupuncturists as it enables them to observe the changes resulting from this treatment in a concrete, timely, and standardized manner. Wen-jia FANG^[18]^ reported a case of acute Bell palsy treated with Fu subcutaneous needling. The researchers used LSCI to observe the improvement of facial blood flow before and after treatment, which provides an idea for the research and clinical practice of Fu subcutaneous needling in the treatment of Bell palsy.

We reported hyperperfusion in the periocular region and the lip area in the healthy controls. When measurement was performed for the corners of the mouth, the lips might have been mistakenly included in the detected area. For this reason, errors in the rate of blood flow changes might be present at the corners of the mouth, which leads to the necessity to exclude the corners of the mouth from the measurement. If only the BP values for the corresponding sites of the face are measured, bilateral differences are usually large and subject to the influence of several factors, including monitoring distance and some diseases leading to facial tissue hyperfusion, such as hypertension and hyperthyroidism. Rather, we estimated the rate of blood flow changes based on bilateral comparison of the corresponding sites. The rate of blood flow changes could more realistically characterize the difference in bilateral facial microcirculatory functions. The influence of baseline facial blood perfusion on the measurements was also reduced by using this method.

Here, the rates of blood flow changes in the periocular region, the cheeks, the corners of the mouth, and the nasolabial fold between the affected and healthy sides were measured by LSCI. These 4 sites of measurement were chosen based on the orientation of the facial nerve exiting the temporal bone, the patients’ main complaints during the treatment, and the sites of abnormal facial muscle movements. In the periocular region and the cheeks, the rates of blood flow changes in patients with Bell palsy and Ramsay Hunt syndrome were significantly decreased when compared with the healthy controls. However, there was no significant difference between patients with Ramsay Hunt syndrome and those with Bell palsy at these sites. We further divided PFP patients into different groups based on severity. The facial blood perfusion decreased on the affected side in both mild and severe PFP. Moreover, the reduction was more significant on the affected side in severe PFP patients than in mild cases. A lower Sunnybrook score was associated with a lower rate of blood flow changes in the periocular region. Thus, the more severe the facial nerve injury, the lower the rate of blood flow changes in the periocular region, and the less the blood perfusion on the affected side would be. These are identical to the results in our previous study.

Facial nerve decompression may be considered for PFP patients who are irresponsive to conservative treatment. We observed a significant increase in blood perfusion in the periocular region after surgery in patients with Bell palsy. There was a fast recovery at about 2 months after surgery, and this trend continued even at 4 months after surgery. However, the blood perfusion in the periocular region did not increase considerably at 2 months after surgery in patients with Ramsay Hunt syndrome. Unexpectedly, the blood perfusion in the periocular region did not increase significantly at 2 months after surgery in patients with Bell palsy, when compared with those with the Ramsay Hunt syndrome. This finding can be interpreted as a significant improvement after surgery in patients with Bell palsy, and in the meantime, the improvement also occurred in patients with the Ramsay Hunt syndrome, though at a slower rate. One reason may be the small number of Ramsay Hunt syndrome patients with whom we conducted follow-up at 4 months after surgery.

In a word, decreased facial blood perfusion was usually observed in PFP patients, and the blood flow changes on the affected side were related to the degree of their facial nerve injury. However, the pattern of facial blood flow changes in PFP did not vary considerably according to etiology (Bell palsy and Ramsay Hunt syndrome). Facial blood perfusion was dramatically improved after surgery in patients with Bell palsy. Although an improvement was also observed in patients with the Ramsay Hunt syndrome, the recovery was slower. The above results confirmed the clinical value of subtotal facial nerve decompression in PFP.

At present, the Sunnybrook score and facial electromyography are usually used to assess the severity of facial nerve injuries. LSCI is a novel technique used to quantify facial blood perfusion, with the following advantages: noninvasive, real-time monitoring, objective and reliable measurements, and ease of operation. In the future, the combination of the Sunnybrook score, facial electromyography, and LSCI may be preferred for assessing the severity of PFP, and for treatment, prognostic prediction, and perioperative recovery guidance.

## 4. Conclusion

PFP patients were usually found to be accompanied by a decrease in facial blood perfusion, and the higher the severity of the facial nerve injury, the lower the facial blood perfusion on the affected side. However, the pattern of facial blood flow changes in PFP did not vary considerably according to etiology (Bell palsy or Ramsay Hunt syndrome). Facial blood perfusion was dramatically improved after surgery in patients with Bell palsy. Although an improvement was also observed in patients with the Ramsay Hunt syndrome, the recovery was slower.

## Author contributions

**Conceptualization:** Jie Wang, Yang Li, Jiandong Li.

**Data curation:** Yang Li, Jiandong Li.

**Formal analysis:** Yang Li, Jiandong Li.

**Writing – original draft:** Jie Wang, Jiandong Li.

**Writing – review & editing:** Jie Wang.
